# Flood impact on men’s mental health: evidence from flood-prone areas of Bangladesh

**DOI:** 10.3389/fpubh.2025.1529558

**Published:** 2025-04-03

**Authors:** Md Mostafizur Rahman, Ifta Alam Shobuj, Md Tanvir Hossain, Edris Alam, Md Kamrul Islam, Md Kaium Hossain

**Affiliations:** ^1^Department of Disaster Management & Resilience, Faculty of Arts and Social Sciences, Bangladesh University of Professionals, Dhaka, Bangladesh; ^2^Sociology Discipline, Social Science School, Khulna University, Khulna, Bangladesh; ^3^Department of Geography and Environmental Studies, University of Chittagong, Chittagong, Bangladesh; ^4^Faculty of Resilience, Rabdan Academy, Abu Dhabi, United Arab Emirates; ^5^Department of Civil and Environmental Engineering, College of Engineering, King Faisal University, Al-Ahsa, Saudi Arabia; ^6^School of Business and Economics, United International University, Dhaka, Bangladesh

**Keywords:** flash flood, mental health, stress, anxiety, depression, Bangladesh

## Abstract

Disasters can pose significant risks to mental health, often resulting in both temporary and long-lasting psychological distress. This study explores the impact of floods on mental health. A survey was conducted shortly after the 2022 flash flood, in which 452 male participants from the Ajmiriganj and Dharmapasha Upazilas in Bangladesh were surveyed. Mental health was assessed using the DASS-21 instrument, and we examined the variables associated with mental health issues. Descriptive statistics and multiple linear regression analysis were employed. Around 47% of participants reported severe or extremely severe depression, 41% reported severe or extremely severe anxiety, and 36% reported severe or extremely severe stress. Factors such as age, marital status, type of home, occupation, flood safety rating, and property loss during the 2022 flood were all found to be associated with depression. Anxiety was linked to flood safety, occupation, housing type, education level, and marital status. Additionally, all anxiety-related variables were also associated with stress. Mental health issues were more prevalent among older, married, illiterate participants living in kacha (temporary) housing, as well as among agricultural workers and fishers with low safety ratings. Psychological interventions and disaster risk reduction strategies could help mitigate the mental health impact of floods. The findings of this study have important implications for global disaster management and public health.

## 1 Introduction

Southeast Asia is highly vulnerable to floods, yet it often lacks the flood-resilient infrastructure necessary to minimize damage and loss (1). Between 1960 and 2015, floods worldwide resulted in the deaths of 35,000 people, with the majority of these fatalities occurring in developing Southeast Asian nations (2). The Ganges Basin’s annual monsoon floods have consistently devastated impoverished, developing countries in South Asia (3). Bangladesh, in particular, is prone to floods, cyclones, droughts, salinity intrusion, landslides, and riverbank erosion (2, 4). The country’s monsoon floods are a regular occurrence, and recent devastating floods in 2017, 2019, 2020, 2021, and 2022 have severely affected its way of life and economy (5–7). The 2017 flood alone affected eight million people, destroying homes, buildings, livestock, and crops (8).

In 2022, northeastern Bangladesh experienced one of the most catastrophic flash floods in recent history (9). The districts of Sylhet and Sunamganj were among the hardest-hit areas, with water levels rising rapidly and submerging entire communities. Ajmiriganj and Dharmapasha Upazilas faced extreme flooding that persisted for several weeks. The flood resulted in massive displacement, extensive damage to homes, loss of agricultural land, and disruptions in transportation and communication. Many residents were left without food, clean drinking water, or access to healthcare, further exacerbating the disaster’s impact on their physical and mental well-being. Given the scale and severity of this flood, understanding its mental health consequences is crucial for informing disaster response strategies.

While the environmental and economic consequences of floods are well-documented, the psychological toll, particularly on mental health, is becoming increasingly evident. Several studies have shown that flood victims are at risk of developing significant mental health issues, including anxiety, stress, depression, and posttraumatic stress disorder (PTSD) (7, 10–15). Factors such as the loss of loved ones, displacement, property damage, crop and agricultural losses, food insecurity, and livelihood disruptions contribute to the mental health challenges faced by flood survivors. In some cases, survivors may also exhibit suicidal tendencies (7, 15). One study found that flood victims experienced nine times higher long-term mental health problems compared to non-flood victims (16). Additionally, rising floodwater levels and a lack of flood warnings have been associated with heightened anxiety, depression, stress, and PTSD (16).

Research has shown that men and women experience different mental health risks following disasters due to variations in societal roles, coping mechanisms, and access to support systems (17). Studies consistently show that women tend to have higher rates of depression, anxiety, and PTSD following disasters. However, men also experience significant psychological distress, often manifesting in externalizing behaviors such as aggression, substance use, and social withdrawal. Societal expectations of masculinity, which discourage emotional expression and help-seeking, can exacerbate men’s mental health struggles post-disaster (18, 19). The traditional perception of masculinity, which emphasizes self-reliance and emotional suppression, often discourages men from seeking psychological support, leading to the accumulation of stress and worsening mental health outcomes (20, 21). Disasters often disrupt livelihoods, financial security, and social roles—factors that disproportionately affect men in patriarchal societies where they are expected to be primary providers (22). While there is evidence that men and women may exhibit different coping strategies under stress, specific studies on gender differences in response to these disasters are limited. Generally, men are often reported to engage in more externalizing behaviors (e.g., substance use, aggression) compared to women, who might experience stress more frequently (23). While studies have consistently found that women are at a higher risk of developing PTSD compared to men after disasters, men also face substantial mental health challenges in these contexts (19, 24). Despite these risks, men are significantly less likely than women to seek professional mental health care post-disaster, which can result in long-term psychological distress (25).

While research on the gendered mental health impacts of disasters in Bangladesh is limited, global studies suggest that men’s mental health challenges post-disaster should not be overlooked (24). Bangladesh is a deeply patriarchal society where traditional gender roles shape expectations for both men and women. Men are typically seen as the primary breadwinners and decision-makers, while women are expected to take on caregiving and domestic responsibilities. These societal norms influence how individuals experience and respond to disasters. During and after a crisis, men face significant pressure to restore financial stability, rebuild homes, and support their families, even when they are experiencing loss and trauma. The expectation of resilience and stoicism discourages men from openly discussing emotional distress or seeking psychological support, which can lead to prolonged mental health challenges.

Several studies show the impact of disaster on men’s mental health. 66% of victims of the 1996 Tangail tornado required psychological support (26). Similarly, 25% of survivors of Cyclone Sidr in 2007 had PTSD, and 18, 16, and 15% experienced depression, somatoform disorder, and mixed anxiety/depressive disorder, respectively (26). In the aftermath of the 2022 floods, many survivors are facing mental health challenges due to economic hardships, which may lead to increased suicide risk (7). Although there are guidelines for mental health care, Bangladesh’s flood mitigation programs lack comprehensive mental health standards, highlighting the need for targeted mental health support and intervention (26). This study aims to fill the gap by examining the psychological distress experienced by men in the aftermath of the 2022 flash flood in Ajmiriganj and Dharmapasha Upazilas of Bangladesh. The study has explored the mental health outcomes of men, who, despite facing unique challenges, have been largely overlooked in post-disaster mental health research. Understanding the gendered experiences of men can help tailor disaster resilience programs and mental health interventions to their specific needs.

This research uses quantitative surveys to study the psychological experiences of men in flood-prone areas. By investigating the mental health effects of the 2022 flood, this research provides crucial insights into how large-scale natural hazards affect men’s psychological well-being. The findings of this study will contribute to developing targeted mental health interventions, improving disaster preparedness, and shaping future policies to address the long-term mental health consequences of such disasters in Bangladesh and beyond.

## 2 Methods

### 2.1 Study design

This study employed a cross-sectional survey design to assess the impact of the 2022 flash flood on men’s mental health in two flood-prone Upazilas of Bangladesh, Ajmiriganj and Dharmapasha. We utilized the Depression, Anxiety, and Stress Scale-21 (DASS-21) to evaluate mental health conditions. A structured questionnaire was administered through face-to-face interviews to collect data on mental health status and associated sociodemographic and flood-related variables. Descriptive statistics and multiple linear regression analyses were applied to examine the association between mental health conditions and various risk factors. Ethical approval for the study was obtained from the Institutional Review Board of Khulna University.

### 2.2 Study area

**Figure 1:**
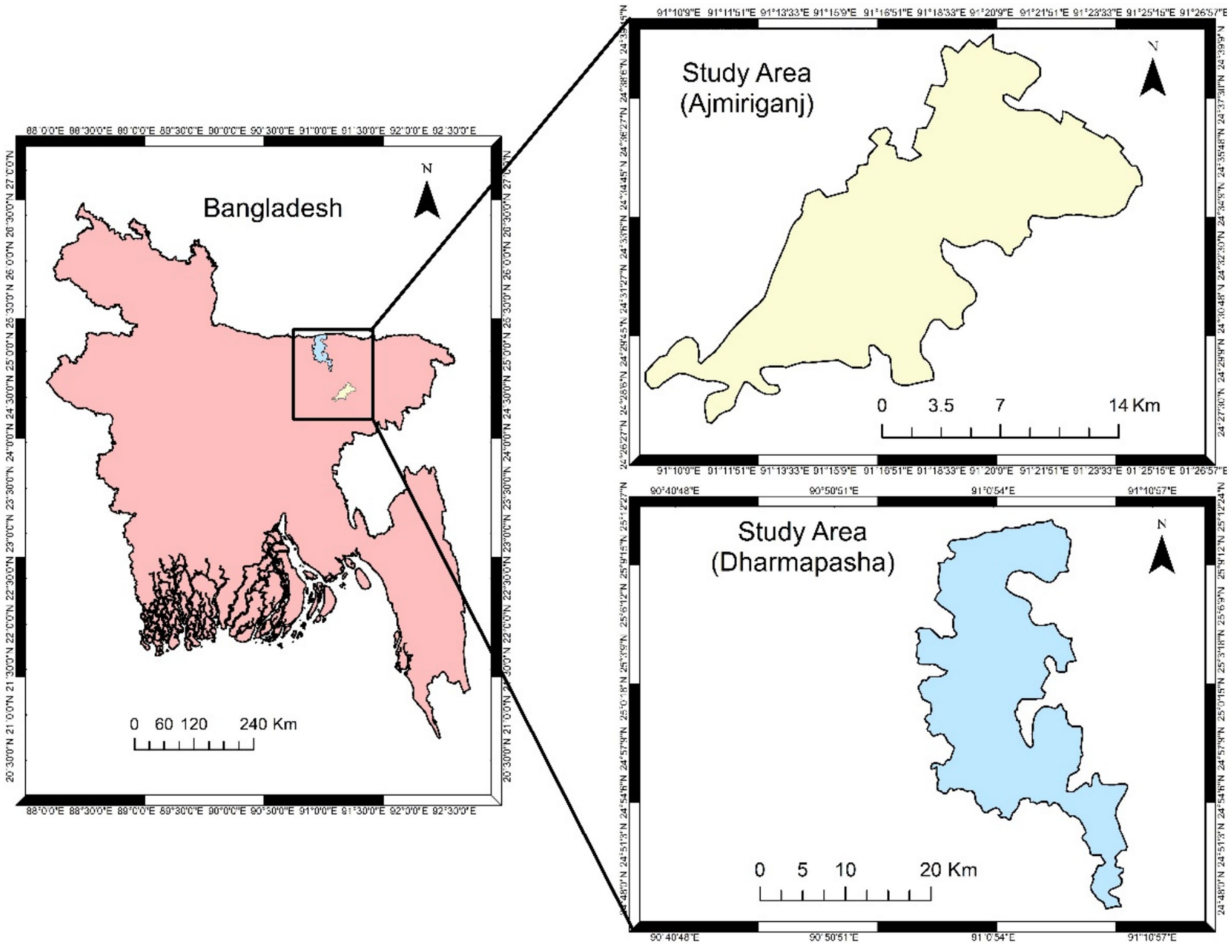
Study area (Source: Authors, 2024).

This cross-sectional study examined the impact of the 2022 floods on two remote Upazilas of Bangladesh: Ajmiriganj and Dharmapasha. Figure 1 shows the locations of these Upazilas, with Ajmiriganj situated in the Habiganj District and Dharmapasha in Sunamganj. The rising water levels of the Khowai, Kushiyara-Kalni rivers, and haors (a type of wetland found in northeastern Bangladesh and parts of India. It is a large, bowl-shaped depression that fills with water during the monsoon season, creating a unique ecosystem. These areas are prone to seasonal flooding, which can have significant impacts on agriculture and livelihoods. Haors are vital for biodiversity, but their floods can be devastating to communities living in or near them) inundating the low-lying areas of Ajmiriganj in Habiganj District. Haors are large, bowl-shaped depressions that fill with water during the monsoon season, creating unique ecosystems. While vital for biodiversity, these areas are prone to seasonal flooding, which can severely impact agriculture and local livelihoods.

In 2022, the floods not only submerged vast areas but also cut off communication and electricity supplies to neighboring areas for several days. The floodwaters severely affected Dharmapasha Upazila, trapping people and damaging road infrastructure. Ajmiriganj Upazila has a population of 114,265, with 56,615 men and 57,650 women, covering a total area of 223.98 km^2^ (27). The male literacy rate in Ajmiriganj is 39.5%, while the female literacy rate is 34.7% for individuals aged seven and older (27). Men in this Upazila primarily work in agriculture, while many women either stay at home or are not employed outside the household. Dharmapasha Upazila has a population of 243,464, with 122,300 men and 121,164 women, and covers an area of 531.00 km^2^ (28). The literacy rate in Dharmapasha is 29.2%, with 30.6% of men and 27.7% of women being literate (27). The majority of residents in both Upazilas rely on agriculture for their livelihood.

The 2022 flash floods in Habiganj District affected 83,390 individuals, with Ajmiriganj being the hardest-hit area (29). The total economic loss in the district was estimated at 5000 million Bangladeshi Taka (approximately 47 million USD), with the most significant losses in infrastructure, education, livestock, agriculture, and fisheries. Most of Ajmiriganj, including its roads, was submerged, leading to a prolonged school closure (30). Similarly, the floods in Dharmapasha submerged around 965 hectares of agricultural land, leading to significant financial losses for local farmers (31). The overall damage to crops in Sunamganj District, including Dharmapasha, amounted to one billion Bangladeshi Taka (approximately nine million USD) (32). Road closures and power disruptions further exacerbated the difficulties faced by flood victims in Dharmapasha Upazila (33).

### 2.3 Survey technique

We conducted a structured survey using face-to-face interviews in the local Bengali language to ensure clarity and comprehension, particularly for illiterate respondents. The questionnaire consisted of closed-ended and Likert-scale questions, including the DASS-21 instrument (34), to assess depression, anxiety, and stress levels. The survey also gathered sociodemographic information, previous flood experiences, and perceptions of flood safety.

The survey was conducted in July–August 2022, after the floodwaters had receded enough to allow access to affected areas. However, some regions were still in recovery, and the flood had a significant impact on the data collection process. Many roads remained damaged or submerged, requiring the research team to use boats and alternative routes to reach certain villages. Some participants were initially hesitant to participate due to their ongoing struggles with property loss, income disruptions, and health concerns. To address these challenges, we collaborated with local community leaders who helped facilitate participant engagement. Despite these difficulties, the survey team successfully conducted in-person interviews, ensuring that responses were gathered from a diverse group of flood-affected individuals.

Seven items comprise each subscale of DASS-21. It was used in various research (35–37). On a four-point Likert scale, 0 means “Did not apply to me at all,” 1 means “Applied to me to some degree, or some of the time-Sometimes,” 2 means “Applied to me to a considerable degree, or a good part of the time-Often,” and 3 means “Applied to me very much or most of the time-Almost always.” Participants had to describe symptoms from the previous week. Add and double the applicable item scores for depression, stress, and anxiety to get the scores. For DASS-21 scores, there are five cutoff points: normal, mild, moderate, severe, and extremely severe (Table 1). The DASS helps measure symptom severity and therapy response.

**Table 1:** Cutoff values for DASS-21 depression, anxiety, and stress labels (34).

Severity label	Depression	Anxiety	Stress
Normal	0–9	0–7	0–14
Mild	10–13	8–9	15–18
Moderate	14–20	10–14	19–25
Severe	21–27	15–19	26–33
Extremely severe	28+	20+	34+

The questionnaire’s final version (in KoboToolbox) incorporates comments from a preliminary survey of certain research participants. Early survey responses were not included in the final study. Cronbach’s alphas are more than 0.80 in all three of the pretested DASS sections, indicating reliability. Current alpha values are close to previous authors’ standards (34). If the value of Cronbach’s alpha exceeds 0.60, then the survey’s internal consistency is considered reliable (38, 39). The questionnaire has four main components. In the first part, we covered demographics (age, marital status, education, location, housing type, occupation, vulnerable family member, and chronic condition). We asked, “How do you perceive your current social life?” concerning social satisfaction. Respondents were questioned in the third part about their past flood experiences prior to the 2022 flood., if their current location was safe from flooding, if they had been injured or ill from the recent flash flood if they had lost a family member, and if they had income problems. This research examined if the flood damaged property. We utilized participant demographics and flood data as independent variables. We anticipated these factors would affect all three DASS components. We then asked DASS-21 questions. Self-reported questionnaires were initially developed (34). Most participants were illiterate or uneducated. We asked questions to get self-reported answers. In certain research, DASS-21 was utilized in face-to-face interviews (40–42). We utilized a tested Bengali form of DASS-21 (43). In our earlier study, we used this tool successfully with the general community during COVID-19 (36). Participants understood our questions. We’ve worked with these participants (44). Our pilot survey also enhanced question clarity.

### 2.4 Data management

Participants for this study were selected using a combination of convenience and snowball sampling techniques. Initially, we approached adult male residents (aged 18 years or older) from Ajmiriganj and Dharmapasha Upazilas who had been directly affected by the 2022 flash flood. The first participant was identified through community contacts and local key informants. Following this, snowball sampling was used, where each respondent was asked to refer other potential participants who met the study criteria. In the convenience sampling technique, participants were selected based on their availability and willingness to participate. This method facilitated quick data collection from those directly affected by the flood. In the case of the snowball sampling technique, initial participants referred other potential respondents from their social networks, ensuring a broader representation of affected individuals. The sample size was determined using Krejcie and Morgan’s (45) table, which provides an established guideline for selecting an appropriate number of respondents. For a population exceeding 10,000 individuals, a sample size of 384 participants is deemed statistically adequate. To enhance reliability and account for potential non-responses, we increased the sample size to 452 participants.

### 2.5 Data analysis

Data analysis was conducted using R software (version 4.2.2) and Python (version 2.7) (46, 47), following a two-step regression approach. First, simple linear regression (bivariate analysis) was performed, where each independent variable was tested separately against the three mental health outcomes—depression, anxiety, and stress—to identify significant associations (*p* < 0.05). In the second step, multiple linear regression (multivariate analysis) was conducted, incorporating only those variables that were statistically significant in the bivariate analysis. Three separate multiple linear regression models were developed, with depression, anxiety, and stress as the respective dependent variables. Each model included selected sociodemographic and flood-related factors as independent variables to assess their associations with mental health conditions. The results were reported using beta coefficients (*β*), confidence intervals (CI), and *p*-values to determine statistical significance.

### 2.6 Ethical issues

An ethical certification committee associated with Khulna University in Bangladesh has approved this research (Ref. No. KUECC-2022/06/16) after reviewing our objectives and method. This study followed the Declaration of Helsinki and its revisions regarding human subject usage (48). Informed consent was obtained from all participants. For those who were illiterate, consent was verbally explained in Bengali, and their agreement was documented with their permission.

## 3 Results and discussion

### 3.1 Sample profile

Table 2 presents the sociodemographic data of the study participants. Approximately 33% of the sample population was between 36 and 55 years old, followed by 24% in the 18–35 age group, with the remaining participants spread across other age groups. The majority (94%) of participants were married. A significant portion of the population was illiterate or had not completed secondary school, which aligns with the low male literacy rates reported in previous Upazila data (27). Our findings, similar to prior research (49), confirm that men in this area tend to have higher education levels than women. Previous studies indicated that 74% of women in these Upazilas are uneducated (49). Most participants lived in semi-pucca dwellings, and more than half of them were employed as agricultural farmers or fishermen.

**Table 2:** Sociodemographic information.

Features	Frequency (%)
1. Age group (year)	
18–35	110 (24.34)
36–45	148 (32.74)
46–55	98 (21.68)
>55	96 (21.24)
2. Marital status	
Married	424 (93.81)
Unmarried	28 (6.19)
3. Education	
Illiterate	208 (46.02)
Non-SSC	208 (46.02)
SSC or above	36 (7.96)
4. Location	
Ajmiriganj	281 (62.17)
Dharmapasha	171 (37.83)
5. Housing type	
Kacha^a^	34 (7.52)
Pucca^b^	18 (3.98)
Semi-pucca	400 (88.50)
6. Occupation	
Agri farmers or Fishers	293 (64.82)
Business	71 (15.71)
Government or private Employee	9 (1.99)
Daily labor	17 (3.76)
Others	11 (2.43)
Unemployed	51 (11.28)
7. Vulnerable family member (child, pregnant woman, older person, etc.)	
Yes	411 (90.93)
No	41 (9.07)
8. Chronic disease	
Maybe	10 (2.21)
No	331 (73.23)
Yes	111 (24.56)
9. Social satisfaction	
Least Satisfied	95 (21.02)
Satisfied	354 (78.32)
Very Satisfied	3 (0.66)

Kacha^a^ = refers to buildings or structures made with temporary or less durable materials such as bamboo, mud, or thatch. These are typically less resistant to floods and other disasters; Pucca^b^ = refers to structures or buildings that are made with durable, permanent materials like brick, concrete, or stone. These buildings are considered more stable and resilient to environmental factors.

Table 3 summarizes the flood-related facts and impacts. A significant number of participants had experienced floods before the 2022 event. Around 55% of participants considered their homes vulnerable to flooding, which is consistent with the regular flooding in these areas (50). They are generally well aware of local flood risks. The 2022 flash flood caused injuries to 13 and 10% of participants’ families. Furthermore, 92% of participants or their families lost income due to the flood, and 95% reported damage to their property. It aligns with a similar report detailing the impacts of the 2022 floods in the region (51). Despite these hardships, most participants received financial and social assistance during the flood event.

**Table 3:** Flood-related information.

Features	Frequency
1. Do you have pre-2022 flood experience?	
No	5 (1.11)
Yes	447 (98.89)
2. How safe is the area from flooding?	
Moderately Safe	189 (41.81)
Safe	13 (2.88)
Unsafe	250 (55.31)
3. Have you been injured or sickened by the 2022 flash flood?	
No	393 (86.95)
Yes	59 (13.05)
4. Do any family members have injuries or diseases from the 2022 flash flood?	
No	405 (89.60)
Yes	47 (10.40)
5. Did you lose any family members in the 2022 flash flood?	
No	446 (98.67)
Yes	6 (1.33)
6. Has the 2022 flash flood harmed your or your family’s income?	
No	38 (8.41)
Yes	414 (91.59)
7. Was your property damaged by the 2022 flash flood?	
No	23 (5.09)
Yes	429 (94.91)
8. Have you obtained social or economic aid during the 2022 flash flood?	
No	66 (14.60)
Yes	386 (85.40)

### 3.2 Mental health status

**Figure 2:**
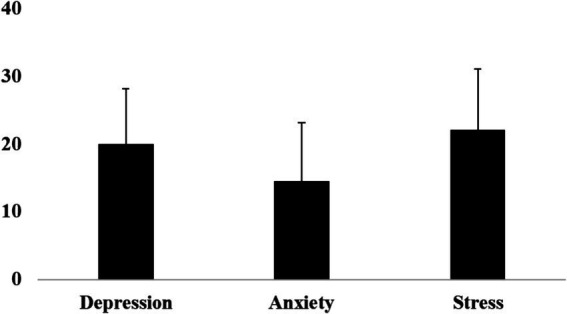
Mean and standard deviation of depression, anxiety, and stress score.

The findings presented in Figure 2 shed light on the mental health status of the research participants, focusing on levels of depression, anxiety, and stress. The mean scores for depression (19.96 ± 8.19), anxiety (14.51 ± 8.6), and stress (22.12 ± 8.98) indicate significant psychological distress among the respondents. Table 4 further highlights the severe psychological impact of the flood. Approximately 24 and 23% of participants experienced severe or extremely severe depression, respectively, pointing to a significant number of individuals struggling with depressive symptoms. Around 33% of participants reported severe anxiety, indicating a high prevalence of anxiety-related distress among men. Additionally, 20 and 16% of respondents experienced severe or extremely severe stress, respectively, further illustrating the widespread stress among participants in the aftermath of the flood.

**Table 4:** Depression, anxiety, and stress labels in men of study areas.

Severity label	Depression [*n* (%)]	Anxiety [*n* (%)]	Stress [*n* (%)]
Normal	53 (11.73)	106 (23.45)	110 (24.34)
Mild	58 (12.83)	50 (11.06)	75 (16.59)
Moderate	130 (28.76)	106 (23.45)	105 (23.23)
Severe	108 (23.89)	42 (9.29)	91 (20.13)
Extremely Severe	103 (22.79)	148 (32.74)	71 (15.71)

These findings are consistent with previous studies on men’s mental health following natural disasters such as floods. Cultural values of stoicism and self-reliance may discourage men from seeking mental health support, potentially exacerbating their suffering (52, 53). The high prevalence of severe mental health symptoms among men underscores the urgent need for targeted mental health treatments and support services in disaster-affected areas.

Men may also experience additional stress due to their societal roles as providers and caretakers. The loss of livelihoods, displacement, or struggles to meet family responsibilities can further strain their mental well-being (53). As a result, men in disaster-affected regions may experience depression, anxiety, stress, and PTSD. Our findings align with broader disaster mental health literature, which suggests that men and women experience psychological distress differently. Women typically exhibit higher rates of PTSD, depression, and anxiety due to heightened emotional processing of trauma. In contrast, men are more likely to manifest stress through externalizing behaviors, such as substance use and avoidance. The high levels of severe depression and anxiety observed in this study reinforce the need for targeted mental health interventions that consider gender-specific coping mechanisms and barriers to seeking psychological support.

A different study on women’s mental health post-disaster found higher levels of depression, anxiety, and stress compared to the results in this study (49). While men may not experience mental health difficulties as severely as women, their challenges also require attention for the sake of community resilience. In countries with entrenched gender equality through legislative frameworks, research suggests that both men and women should face similar disaster-related mental health outcomes (54). For instance, the 2007 Tewkesbury floods and the 2008 Morpeth floods in the UK demonstrated that men and women may experience both common and distinct gendered impacts from disasters (54). Similarly, the English National Study on Flooding and Health found that women had comparable risks of depression and anxiety as men (55). After Hurricane Katrina, 15% of men in the affected areas reported depression (56). In some cases, men expressed feelings of fear and referred to the floods as “very severe” (54).

### 3.3 Associated factors with depression, anxiety, and stress

Table 5 outlines the Disaster-Related Adjustment and Stress (DAS) factors. In line with the method used in this study, only statistically significant variables were included in the simple linear regression analysis. Significant variables included age group, education, marital status, housing type, occupation, chronic disease, social satisfaction, prior flood experience, flood safety ratings, income loss due to the flood, property damage in the 2022 flood, and receiving social and economic support during the flood. Multiple linear regression analyses indicated that depression was significantly associated with age, marital status, housing type, occupation, flood safety ratings, and property damage in the 2022 flood.

**Table 5:** Associated factors with DAS.

Features		β^#^ (95% CI)		
		Model I Depression	Model II Anxiety	Model III Stress
1. Age range (in year)	18–35	−1.26 (−3.49; 0.95)	−0.74 (−3.14; 1.64)	−0.64 (−3.16; 1.87)
	36–45	−2.02 (−4.02; −0.02)*	−0.65 (−2.79; 1.47)	−0.89 (−3.14; 1.35)
	46–55	−1.11 (−3.21; 0.97)	−0.42 (−2.64; 1.80)	0.66 (−1.67; 3.01)
	>55			
2. Marital status	Married			
	Unmarried	−4.73 (−7.66; −1.80)**	−4.01 (−7.16; −0.87)*	−5.06 (−8.36; −1.76)**
3. Education	Illiterate			
	Non-SSC	−0.47 (−1.92; 0.97)	−1.80 (−3.43; −0.17)*	−1.84 (−3.55; −0.14)*
	SSC or above	−0.35 (−3.02; 2.31)	−1.19 (−4.08; 1.69)	−1.50 (−4.51; 1.51)
4. Location	Ajmiriganj			
	Dharmapasha		−0.32 (−2.08; 1.42)	1.53 (−0.30; 3.37)
5. Types of housing structure	Kacha			
	Pucca	−6.74 (−10.83; −2.64)**	−6.58 (−10.98; −2.18)*	−5.59 (−10.18; −1.00)*
	Semi-pucca	−0.35 (−2.73; 2.01)	−2.40 (−4.95; 0.14)	−0.62 (−3.28; 2.03)
6. Occupation	Agri farmers or fishers			
	Business	−3.74 (−5.55; −1.93)***	−2.42 (−4.37; −0.46)*	−4.19 (−6.23; −2.15)***
	Government or private employee	−5.84 (−11.67; −0.01)*	−5.33 (−10.74; 0.08)	−7.91 (−14.35; −1.47)*
	Daily labor	−0.78 (−4.08; 2.50)	−2.17 (−5.77; 1.41)	−1.98 (−5.73; 1.75)
	Others	−1.86 (−6.03; 2.30)	0.85 (−3.66; 5.37)	−1.56 (−6.28; 3.15)
	Unemployed	1.25 (−1.30; 3.81)	0.30 (−2.41; 3.03)	−3.97 (−7.09; −0.84)*
7. Chronic disease	Maybe			
	No	0.48 (−3.80; 4.77)	1.61 (−3.00; 6.23)	1.33 (−3.52; 6.19)
	Yes	1.97 (−2.48; 6.43)	4.02 (−0.80; 8.85)	1.83 (−3.21; 6.88)
8. Social satisfaction	Least satisfied			
	Satisfied	−1.17 (−2.74; 0.40)	0.25 (−1.43; 1.94)	−0.46 (−2.22; 1.29)
	Very satisfied	1.61 (−6.44; 9.68)	−1.37 (−9.82; 7.07)	−2.91 (−11.96; 6.12)
9. Do you have pre-2022 flood experience?	No			
	Yes	−0.62 (−6.87; 5.61)		
10. How safe is the area from flooding?	Moderately safe			
	Safe	−0.50 (−4.55; −3.54)	1.44 (−2.90; 5.79)	1.36 (−3.20; 5.93)
	Unsafe	5.99 (4.63; 7.35)***	6.55 (5.03; 8.08)***	7.05 (5.45; 8.65)***
11. Has the 2022 flash flood harmed your or your family’s income?	No			
	Yes	−0.53 (−3.36; 2.30)		−0.36 (−3.56; 2.82)
12. Was your property damaged by the 2022 flash flood?	No			
	Yes	4.29 (1.16; 7.41)**		2.35 (−1.13; 5.84)
13. Have you obtained social or economic aid during the 2022 flash flood?	No			
	Yes	0.04 (−1.90; 1.98)	1.30 (−0.79; 3.40)	0.23 (−1.97; 2.45)

*p < 0.05, **p < 0.01, ***p < 0.001; β^#^ = Beta (Coefficient). CI, confidence interval.

Participants in the 36–45 age group, unmarried individuals, those living in pucca (brick) houses, those engaged in business activities or employed in government or private sectors, and individuals who rated their areas as moderately safe from flooding and had not suffered property damage were less likely to experience depression than those in the over-55 age group, married individuals, those residing in kacha (temporary) houses, and those working as fishermen or farmers, particularly if they perceived their areas as unsafe from floods or had experienced property damage in previous floods, including the 2022 event.

Anxiety was found to be associated with marital status, education, housing type, occupation, and flood safety ratings. Unmarried participants, those with less than secondary school education, those living in pucca houses, businesspeople, and those who rated their areas as moderately safe from flooding reported lower levels of anxiety compared to married individuals, those with no formal education, those residing in kacha houses, and those working in agriculture or fishing, particularly in areas rated as unsafe.

Stress was associated with all anxiety-related factors. Unmarried participants, those with less than secondary education, those living in pucca houses, businesspeople, government or private employees, and those living in moderately safe locations were less likely to experience stress compared to married individuals, those with no formal education, those residing in kacha houses, and those involved in agriculture or fishing, particularly in unsafe areas.

The impact of sociodemographic factors on mental health post-floods is clear. These factors significantly affect mental health outcomes in the aftermath of disasters. Floods, due to their recurring nature and severity, can exacerbate mental health problems, especially among vulnerable groups, such as people with low incomes (57). These communities, often dependent on agriculture or fishing, suffer significant losses of livelihoods, which compounds mental health distress. Flooding is one of the most damaging agricultural disasters, leading to crop failure and decreased productivity (58, 59). It also disrupts water quality and habitat structures, which adversely affect fishing (60, 61). Thus, the mental health challenges observed in our study can be attributed to the destruction of livelihoods caused by the 2022 floods.

Identifying these key sociodemographic characteristics allows authorities, public health professionals, and disaster management experts to better target interventions for vulnerable populations. Understanding the root causes of mental health disorders is crucial. Older individuals may have experienced multiple disasters over time, leading to increased mental health concerns (62, 63). Older people are particularly vulnerable to the impacts of disasters, often facing increased risks due to diminished physical health and social isolation (64, 65). Research suggests that older adults are more likely to suffer from mental health problems in the wake of a disaster (66–69).

The DAS factors also reveal the role of inadequate education, housing quality, and the safety of living environments in shaping mental health outcomes. Mental illness places a heavy burden on individuals, families, and communities (70). Low socioeconomic status is linked to higher rates of mental health issues (71). The mismatch between demands and available resources often triggers stress responses. People with low incomes face greater health risks yet lack the resources to mitigate them (72). Limited response resources in poor communities increase vulnerability to stress, conflict, and hazardous conditions (73), leading to long-term mental health consequences.

Illiterate individuals may be less aware of disaster risks and less able to respond effectively (74). Studies show that education plays a critical role in enhancing resilience and mental health post-disasters (75–77). Nations with higher levels of income and education typically experience fewer losses during disasters (78). It raises questions about whether financial stress contributes to mental health problems in low-income populations or whether education plays a more significant role in fostering resilience (79). Education can improve awareness of disaster risks, disaster preparedness, and access to resources (75). In contrast, inadequate disaster response, especially in flood-prone areas, may lead to increased mental health symptoms. Different regions experience varying levels of flood damage depending on housing quality and the vulnerability of exposed elements (80). Participants living in kacha houses, for example, were found to have higher levels of depression, anxiety, and stress compared to those living in pucca houses, likely due to the hazardous conditions associated with kacha housing.

Overall, our findings demonstrate that the mental health issues experienced by participants are directly related to the losses suffered during the 2022 floods. As loss from disasters can exacerbate mental health challenges, it is crucial to prioritize both disaster risk mitigation and post-disaster mental health care. The mental health impacts of floods are significantly influenced by community resilience. Resilient communities tend to experience fewer mental health problems post-flood (81). Several factors, such as traumatic events, disruptions to daily life, the loss of loved ones, and the destruction of homes and assets, can contribute to the increase in mental health issues (82).

### 3.4 Recommendations

Based on our findings, we propose the following recommendations for local and national governments, as well as disaster management and public health authorities, to mitigate the mental health impact of floods:

#### 3.4.1 Strengthening mental health support in disaster response

Mental health services should be integrated into emergency response programs, focusing on high-risk groups identified in this study, including older individuals, those in unsafe housing, and agricultural workers. Healthcare workers and community volunteers should receive training in Psychological First Aid (PFA) to provide immediate post-disaster mental health support. Additionally, public awareness campaigns should be expanded to reduce the stigma surrounding mental health issues among men, encouraging them to seek help when needed.

#### 3.4.2 Targeted mental health interventions

Specialized mental health programs should be developed for flood-affected men, particularly those with lower education levels and precarious livelihoods. Access to counseling and psychosocial support services should be enhanced through mobile health clinics, especially in remote flood-prone areas where traditional healthcare access is limited. These interventions should be tailored to address the unique psychological challenges faced by men in disaster settings.

#### 3.4.3 Improving flood preparedness and housing resilience

Early warning systems should be strengthened to ensure timely flood alerts, reducing uncertainty and psychological distress among vulnerable populations. In addition, housing improvement programs should be implemented to support the transition from kacha (temporary) housing to more resilient structures, thereby mitigating future mental health risks associated with displacement and property loss.

#### 3.4.4 Enhancing socioeconomic recovery programs

Targeted financial aid and livelihood recovery initiatives should be provided to support flood-affected men, particularly those in high-risk occupations. Microfinance and vocational training programs should be promoted to diversify income sources and reduce economic vulnerabilities. Ensuring financial stability post-disaster can play a critical role in reducing long-term psychological distress and supporting mental health recovery.

#### 3.4.5 Integrating mental health into disaster policy

Mental health considerations should be incorporated into Bangladesh’s national disaster risk reduction strategies to ensure comprehensive disaster response planning. Additionally, long-term mental health monitoring programs should be established in flood-prone areas to track and address persistent psychological effects. This approach will help policymakers and practitioners develop effective strategies to mitigate mental health challenges in future disaster events.

## 4 Strengths and limitations

This study successfully assessed the prevalence and associated factors of depression, anxiety, and stress among flood-affected men, identifying statistical associations between flood exposure and mental health outcomes. However, the research has certain limitations. The use of convenience and snowball sampling may have introduced selection bias, and the data collection timeframe was predefined, restricting broader generalization. The cross-sectional design captures associations at a single time point but does not establish causal relationships between flooding and mental health distress. Additionally, due to widespread illiteracy, only straightforward inquiries were made. The study focused solely on male casualties of the 2022 flash flood, excluding men who remained unharmed. Despite these limitations, this baseline survey provides valuable insights for ongoing research on disaster-related mental health concerns. Future longitudinal studies could help confirm the long-term psychological impact of floods. Moreover, the study’s findings can inform disaster risk reduction strategies, aiding officials in developing more effective flood recovery and preparedness measures. Similar methodologies may also be applied to assess the mental health impacts of natural hazards across different regions, both nationally and internationally.

## 5 Conclusion

This study investigates the impact of the 2022 flash floods on men’s mental health in two severely affected Upazilas of Bangladesh. Using the DASS-21 scale, we assessed the prevalence of depression, anxiety, and stress among flood survivors. Our findings indicate that a significant proportion of respondents experienced severe psychological distress, with sociodemographic and flood-related factors playing a crucial role. Older age, lower education levels, unsafe housing conditions, and employment in agriculture or fishing were associated with higher mental health burdens. The study underscores the importance of addressing mental health challenges in disaster response and preparedness efforts. While the findings provide valuable insights, they also highlight the need for further research, particularly longitudinal studies, to assess long-term psychological impacts. Additionally, the study’s reliance on self-reported data and cross-sectional design limits causal interpretations. Future research should explore gender-specific coping strategies and resilience factors to better inform mental health interventions in disaster-prone areas.

## Data Availability

The raw data supporting the conclusions of this article will be made available by the authors, without undue reservation.

## References

[1] Middleton C Elmhirst R Chantavanich S. Living with floods in a Mobile Southeast Asia Taylor & Francis (2017).

[2] UNISDR C. The human cost of natural disasters: a global perspective. (2015)

[3] Priya S Young W Hopson T Avasthi A. Planning for disaster: forecasting the impact of floods in South Asia's river basins. Plan Disaster Forecast Impact Floods South Asias River Basins (2017). Available online at: https://blogs.worldbank.org/water/planning-disaster-forecasting-impact-floods-south-asias-river-basins (accessed January 20, 2023)

[4] Juran L Trivedi J. Women, gender norms, and natural disasters in Bangladesh. Geogr Rev. (2015) 105:601–11. doi: 10.1111/j.1931-0846.2015.12089.x

[5] Das TK. Fighting floods for survival: experiences of suffering people in Bangladesh In: Singh RB Prokop P, editors. Environmental geography of South Asia. Advances in geographical and environmental sciences. Tokyo: Springer Japan (2016). 335–51.

[6] Haque A Jahan S. Impact of flood disasters in Bangladesh: a multi-sector regional analysis. Int J Disaster Risk Reduct. (2015) 13:266–75. doi: 10.1016/j.ijdrr.2015.07.001, PMID: 40128009

[7] Mamun MA Safiq MB Hosen I Mamun F. Suicidal behavior and flood effects in Bangladesh: a two-site interview study. Risk Manag Healthc Policy. (2021) 14:129–42. doi: 10.2147/RMHP.S282965, PMID: 33469396 PMC7812054

[8] Dey NC Parvez M Islam MR. A study on the impact of the 2017 early monsoon flash flood: potential measures to safeguard livelihoods from extreme climate events in the haor area of Bangladesh. Int J Disaster Risk Reduct. (2021) 59:102247. doi: 10.1016/j.ijdrr.2021.102247, PMID: 40128009

[9] Hossain E. Second wave of flash flood hits Sylhet haor. (2022). Available online at: https://www.newagebd.net/article/168191/second-wave-of-flash-flood-hits-sylhet-haor (accessed July 29, 2022)

[10] An R Qiu Y Xiang X Ji M Guan C. Impact of hurricane Katrina on mental health among US adults. Am J Health Behav. (2019) 43:1186–99. doi: 10.5993/AJHB.43.6.15, PMID: 31662176

[11] Brooks SK Dunn R Amlôt R Greenberg N Rubin GJ. Training and post-disaster interventions for the psychological impacts on disaster-exposed employees: a systematic review. J Ment Health. (2018):1–25. doi: 10.1080/09638237.2018.1437610, PMID: 29447058

[12] Graham H White P Cotton J McManus S. Flood- and weather-damaged homes and mental health: an analysis using England's mental health survey. Int J Environ Res Public Health. (2019) 16:3256. doi: 10.3390/ijerph16183256, PMID: 31491859 PMC6765946

[13] Makwana N. Disaster and its impact on mental health: a narrative review. J Fam Med Prim Care. (2019) 8:3090–5. doi: 10.4103/jfmpc.jfmpc_893_19, PMID: 31742125 PMC6857396

[14] Manove EE Lowe SR Bonumwezi J Preston J Waters MC Rhodes JE. Posttraumatic growth in low-income black mothers who survived hurricane Katrina. Am J Orthopsychiatry. (2019) 89:144–58. doi: 10.1037/ort0000398, PMID: 30676050 PMC6666311

[15] Parida Y Dash DP Bhardwaj P Chowdhury JR. Effects of drought and flood on farmer suicides in Indian states: An empirical analysis. Econ Disasters Clim Change. (2018) 2:159–80. doi: 10.1007/s41885-018-0023-8

[16] Cruz J White PCL Bell A Coventry PA. Effect of extreme weather events on mental health: a narrative synthesis and Meta-analysis for the UK. Int J Environ Res Public Health. (2020) 17:8581. doi: 10.3390/ijerph17228581, PMID: 33227944 PMC7699288

[17] First JM Bonifay W Houston JB. Gender differences in posttraumatic stress symptoms after a disaster: a differential item functioning analysis of the impact of event scale-revised. J Soc Soc Work Res. (2021) 12:657–76. doi: 10.1086/717263

[18] Cooks-Campbell A. Men's mental health: Why resilience is bigger than invulnerability. (2021). Available online at: https://www.betterup.com/blog/mens-mental-health (accessed March 16, 2025)

[19] Yazawa A Aida J Kondo K Kawachi I. Gender differences in risk of posttraumatic stress symptoms after disaster among older people: differential exposure or differential vulnerability? J Affect Disord. (2022) 297:447–54. doi: 10.1016/j.jad.2021.10.094, PMID: 34715197 PMC8629870

[20] Oliffe JL Ogrodniczuk JS Bottorff JL Johnson JL Hoyak K. “You feel like you can't live anymore”: suicide from the perspectives of Canadian men who experience depression. Soc Sci Med. (2012) 74:506–14. doi: 10.1016/j.socscimed.2010.03.057 20541308

[21] Sileo KM Kershaw TS. Dimensions of masculine norms, depression, and mental health service utilization: results from a prospective cohort study among emerging adult men in the United States. Am J Mens Health. (2020) 14:1557988320906980. doi: 10.1177/1557988320906980, PMID: 32079448 PMC7036518

[22] Enarson E Pease B. The gendered terrain of disaster: thinking about men and masculinities In:. Men, masculinities and disaster: Routledge (2016)

[23] Moise IK. Hospitalizations for substance abuse disorders before and after hurricane Katrina: spatial clustering and area-level predictors, New Orleans, 2004 and 2008. Prev Chronic Dis. (2016) 13:107. doi: 10.5888/pcd13.160107, PMID: 27736053 PMC5063608

[24] Sohrabizadeh S Eslami A. Men's health in disasters: a systematic scoping review. J Iran Med Counc. (2025) 8:3–12. doi: 10.18502/jimc.v8i1.17055, PMID: 39906092

[25] Lee J Trudel R. Man up! The mental health-feminine stereotype and its effect on the adoption of mental health apps. J Consum Psychol. (2025) 35:121–8. doi: 10.1002/jcpy.1405

[26] Nahar N Blomstedt Y Wu B Kandarina I Trisnantoro L Kinsman J. Increasing the provision of mental health care for vulnerable, disaster-affected people in Bangladesh. BMC Public Health. (2014) 14:708. doi: 10.1186/1471-2458-14-708, PMID: 25011931 PMC4099388

[27] Bangladesh Bureau of Statistics (2011). Available online at: http://www.bbs.gov.bd/site/page/2888a55d-d686-4736-bad0-54b70462afda/District-Statistics (accessed October 14, 2022)

[28] Upazila D. Bloggingorg Blog (2023). Available online at: http://dharmapasha.sunamganj.gov.bd/en/site/page/O8lL-%E0%A6%8F%E0%A6%95-%E0%A6%A8%E0%A6%9C%E0%A6%B0%E0%A7%87 (accessed March 30, 2023)

[29] Islam MS. Flood causes loss of 500C in Habiganj. Dhaka Trib. Available online at: https://www.dhakatribune.com/bangladesh/290142/flood-causes-loss-of-500c-in-habiganj (accessed August 3, 2023)

[30] Hasan. Flood situation worsens in Habiganj, academic activities being disturbed. Risingbd Online Bangla News Portal (2022). Available online at: https://www.risingbd.com/english/country/news/87516 (accessed March 30, 2023)

[31] Flash flood damages 5,000 hectares of Boro crops in Sunamganj haors. Bus Stand (2022). Available online at: https://www.tbsnews.net/economy/flash-flood-damages-5000-hectares-boro-crops-sunamganj-haors-400562 (accessed March 31, 2023)

[32] Flash floods damage crops worth Tk 1.0b in Sunamganj. Financ Express (2022). Available online at: https://thefinancialexpress.com.bd/national/flash-floods-damage-crops-worth-tk-10b-in-sunamganj-1649506117 (accessed March 31, 2023)

[33] Chowdhury D. 40 lakh people stranded in Sylhet, Sunamganj flood. Dly Star (2022). Available online at: https://www.thedailystar.net/environment/climate-crisis/natural-disaster/news/40-lakh-people-stranded-sylhet-sunamganj-floodwater-3050221 (accessed March 31, 2023)

[34] Lovibond PF Lovibond SH. The structure of negative emotional states: comparison of the depression anxiety stress scales (DASS) with the Beck depression and anxiety inventories. Behav Res Ther. (1995) 33:335–43. doi: 10.1016/0005-7967(94)00075-U, PMID: 7726811

[35] Hossain A Alam MJ Haque MR. Effects of riverbank erosion on mental health of the affected people in Bangladesh. PLoS One. (2021) 16:e0254782. doi: 10.1371/journal.pone.0254782, PMID: 34292997 PMC8297774

[36] Rahman MM Khan SJ Sakib MS Chakma S Procheta NF Mamun ZA. Assessing the psychological condition among general people of Bangladesh during COVID-19 pandemic. J Hum Behav Soc Environ. (2020) 31:449–63. doi: 10.1080/10911359.2020.1848688, PMID: 40101104

[37] Rahman MM Amin T Sultan SB Bithi MI Rahman F Rahman MM. Depression, anxiety, and stress among public university students in Bangladesh during the COVID-19 pandemic. J Emerg Manag. (2021) 19:99–107. doi: 10.5055/jem.061634723371

[38] Ursachi G Horodnic IA Zait A. How reliable are measurement scales? External factors with indirect influence on reliability estimators. Procedia Econ Finance. (2015) 20:679–86. doi: 10.1016/S2212-5671(15)00123-9

[39] Radhakrishna RB. Tips for developing and testing questionnaires/instruments. J Ext. (2007) 45:1–4.

[40] Bener A Gerber LM Sheikh J. Prevalence of psychiatric disorders and associated risk factors in women during their postpartum period: a major public health problem and global comparison. Int J Women's Health. (2012) 4:191–200. doi: 10.2147/IJWH.S29380, PMID: 22654524 PMC3363135

[41] Le TA Le MQT Dang AD Dang AK Nguyen CT Pham HQ. Multi-level predictors of psychological problems among methadone maintenance treatment patients in difference types of settings in Vietnam. Subst Abuse Treat Prev Policy. (2019) 14:39. doi: 10.1186/s13011-019-0223-4, PMID: 31533764 PMC6751619

[42] Mohammad KI Gamble J Creedy DK. Prevalence and factors associated with the development of antenatal and postnatal depression among Jordanian women. Midwifery. (2011) 27:e238–45. doi: 10.1016/j.midw.2010.10.008, PMID: 21130548

[43] Alim SAHM Kibria SME Uddin MZ Nessa M Wahab MA. Translation of DASS 21 into Bangla and validation among medical students. Bangladesh J Psychiatry. (2014) 28:67–70. doi: 10.3329/bjpsy.v28i2.32740

[44] Rahman MM Shobuj IA Santo MMH Hossain MT. Public perception toward lightning in a lightning-prone area of Bangladesh. Int J Disaster Risk Reduct. (2023) 89:103638. doi: 10.1016/j.ijdrr.2023.103638, PMID: 40128009

[45] Krejcie RV Morgan DW. Determining sample size for research activities. Educ Psychol Meas. (1970) 30:607–10. doi: 10.1177/001316447003000308

[46] R Development Core Team. R: A language and environment for statistical computing, version 4.2.2. Vienna, Austria: R Foundation for Statistical Computing (2022).

[47] Welcome to Python.org. Python.Org. Available online at: https://www.python.org/ (accessed January 15, 2021)

[48] WMA – The world medical association-WMA declaration of Helsinki – ethical principles for medical research involving human subjects. Available online at: https://www.wma.net/policies-post/wma-declaration-of-helsinki-ethical-principles-for-medical-research-involving-human-subjects/ (accessed August 9, 2021)

[49] Rahman MM Alam Shobuj I Tanvir Hossain M Tasnim F. Impact of disaster on mental health of women: a case study on 2022 flash flood in Bangladesh. Int J Disaster Risk Reduct. (2023) 96:103935. doi: 10.1016/j.ijdrr.2023.103935, PMID: 40128009

[50] Kamruzzaman M Shaw R. Flood and sustainable agriculture in the Haor basin of Bangladesh: a review paper. Univers J Agric Res. (2018) 6:40–9. doi: 10.13189/ujar.2018.060106

[51] Flash flood in northeastern Bangladesh (Sunamganj, Moulavibazar and Netrokona district), briefing note – 02/08/2022 – Bangladesh | ReliefWeb. (2022). Available online at: https://reliefweb.int/report/bangladesh/flash-flood-northeastern-bangladesh-sunamganj-moulavibazar-and-netrokona-district-briefing-note-02082022 (accessed April 3, 2023)

[52] Addis ME Mahalik JR. Men, masculinity, and the contexts of help seeking. Am Psychol. (2003) 58:5–14. doi: 10.1037/0003-066X.58.1.5, PMID: 12674814

[53] Oliffe JL Phillips MJ. Men, depression and masculinities: a review and recommendations. J Mens Health. (2008) 5:194–202. doi: 10.1016/j.jomh.2008.03.016

[54] Akerkar S Fordham M. Gender, place and mental health recovery in disasters: addressing issues of equality and difference. Int J Disaster Risk Reduct. (2017) 23:218–30. doi: 10.1016/j.ijdrr.2017.03.014

[55] Tempest EL English National Study on Flooding and Health Study Group Carter B Beck CR Rubin GJ. Secondary stressors are associated with probable psychological morbidity after flooding: a cross-sectional analysis. Eur J Pub Health. (2017) 27:1042–7. doi: 10.1093/eurpub/ckx182, PMID: 29087460 PMC5881756

[56] Galea S Brewin CR Gruber M Jones RT King DW King LA. Exposure to hurricane-related stressors and mental illness after hurricane Katrina. Arch Gen Psychiatry. (2007) 64:1427–34. doi: 10.1001/archpsyc.64.12.1427, PMID: 18056551 PMC2174368

[57] Matthews V Longman J Berry HL Passey M Bennett-Levy J Morgan GG. Differential mental health impact six months after Extensive River flooding in rural Australia: a cross-sectional analysis through an equity Lens. Front Public Health. (2019) 7:367. doi: 10.3389/fpubh.2019.00367, PMID: 31867302 PMC6909816

[58] Dang Y Yang L Song J. The construction of a crop flood damage assessment index to rapidly assess the extent of Postdisaster impact. Remote Sens. (2024) 16:1527. doi: 10.3390/rs16091527

[59] Shah SA Ai S. Flood susceptibility mapping contributes to disaster risk reduction: a case study in Sindh, Pakistan. Int J Disaster Risk Reduct. (2024) 108:104503. doi: 10.1016/j.ijdrr.2024.104503

[60] Talbot CJ Bennett EM Cassell K Hanes DM Minor EC Paerl H. The impact of flooding on aquatic ecosystem services. Biogeochemistry. (2018) 141:439–61. doi: 10.1007/s10533-018-0449-7, PMID: 30930510 PMC6404734

[61] Borah BC. Impact of climate change-induced challenges on fisheries in the north eastern region of India and the way ahead. Aquat Ecosyst Health Manag. (2021) 24:94–102. doi: 10.14321/aehm.024.03.11

[62] Bolin R Klenow DJ. Response of the elderly to disaster: an age-stratified analysis. Int J Aging Hum Dev. (1983) 16:283–96. doi: 10.2190/MQEG-YN39-8D5V-WKMP, PMID: 7184870

[63] Choudhury WA Quraishi FA Haque Z. Mental health and psychosocial aspects of disaster preparedness in Bangladesh. Int Rev Psychiatry. (2006) 18:529–35. doi: 10.1080/09540260601037896, PMID: 17162693

[64] Lee H-K Hong W-H Lee Y-H. Experimental study on the influence of water depth on the evacuation speed of elderly people in flood conditions. Int J Disaster Risk Reduct. (2019) 39:101198. doi: 10.1016/j.ijdrr.2019.101198, PMID: 40128009

[65] Meyer MA. Elderly perceptions of social capital and age-related disaster vulnerability. Disaster Med Public Health Prep. (2017) 11:48–55. doi: 10.1017/dmp.2016.139, PMID: 27839520

[66] Geller A. The susceptibility of older adults to environmental hazards. Generations. (2009) 33:10–8.

[67] Heller K Alexander DB Gatz M Knight BG Rose T. Social and personal factors as predictors of earthquake preparation: the role of support provision, network discussion, negative affect, age, and Education1. J Appl Soc Psychol. (2005) 35:399–422. doi: 10.1111/j.1559-1816.2005.tb02127.x

[68] Malak MA Sajib AM Quader MA Anjum H. “We are feeling older than our age”: vulnerability and adaptive strategies of aging people to cyclones in coastal Bangladesh. Int J Disaster Risk Reduct. (2020) 48:101595. doi: 10.1016/j.ijdrr.2020.101595

[69] Ngo EB. When disasters and age collide: reviewing vulnerability of the elderly. Nat Hazards Rev. (2001) 2:80–9. doi: 10.1061/(ASCE)1527-6988(2001)2:2(80)

[70] Gabriel P Liimatainen M-R. Mental health in the workplace: introduction, executive summaries. (2000). Available online at: https://ecommons.cornell.edu/server/api/core/bitstreams/86b68b74-b3e1-4ee1-85bd-502177a538cd/content (accessed March 6, 2023)

[71] Lorant V Deliège D Eaton W Robert A Philippot P Ansseau M. Socioeconomic inequalities in depression: a meta-analysis. Am J Epidemiol. (2003) 157:98–112. doi: 10.1093/aje/kwf182, PMID: 12522017

[72] Lazarus RS. Stress and emotion: a new synthesis Springer Publishing Company (2006).

[73] Adler NE Rehkopf DHUS. Disparities in health: descriptions, causes, and mechanisms. Annu Rev Public Health. (2008) 29:235–52. doi: 10.1146/annurev.publhealth.29.020907.090852 18031225

[74] Sun R Gong Z Gao G Shah AA. Comparative analysis of multi-criteria decision-making methods for flood disaster risk in the Yangtze River Delta. Int J Disaster Risk Reduct. (2020) 51:101768. doi: 10.1016/j.ijdrr.2020.101768

[75] Chen S Bagrodia R Pfeffer CC Meli L Bonanno GA. Anxiety and resilience in the face of natural disasters associated with climate change: a review and methodological critique. J Anxiety Disord. (2020) 76:102297. doi: 10.1016/j.janxdis.2020.102297, PMID: 32957002

[76] Mandavia AD Bonanno GA. When natural disaster follows economic downturn: the incremental impact of multiple stressor events on trajectories of depression and posttraumatic stress disorder. Disaster Med Public Health Prep. (2019) 13:173–82. doi: 10.1017/dmp.2018.12, PMID: 29704903

[77] Norris FH Friedman MJ Watson PJ Byrne CM Diaz E Kaniasty K. 60,000 disaster victims speak: part I. An empirical review of the empirical literature, 1981–2001. Psychiatry Interpers Biol Process. (2002) 65:207–39. doi: 10.1521/psyc.65.3.207.20173, PMID: 12405079

[78] Weems CF. The importance of the post-disaster context in fostering human resilience. Lancet Planet Health. (2019) 3:e53–4. doi: 10.1016/S2542-5196(19)30014-2, PMID: 30797404

[79] Saeed SA Gargano SP. Natural disasters and mental health. Int Rev Psychiatry. (2022) 34:16–25. doi: 10.1080/09540261.2022.2037524 35584023

[80] Shrestha BB Kawasaki A Zin WW. Development of flood damage assessment method for residential areas considering various house types for Bago Region of Myanmar. Int J Disaster Risk Reduct. (2021) 66:102602. doi: 10.1016/j.ijdrr.2021.102602, PMID: 40128009

[81] Dewa O Makoka D Ayo-Yusuf OA. Measuring community flood resilience and associated factors in rural Malawi. J Flood Risk Manag. (2023) 16:e12874. doi: 10.1111/jfr3.12874

[82] Morganstein JC Ursano RJ. Ecological disasters and mental health: causes, consequences, and interventions. Front Psych. (2020) 11:1. doi: 10.3389/fpsyt.2020.00001, PMID: 32116830 PMC7026686

